# RELIEF registry: large-scale evidence on the safety and long-term efficacy of focused ultrasound treatment of symptomatic leiomyoma

**DOI:** 10.1186/2050-5736-3-S1-O94

**Published:** 2015-06-30

**Authors:** Arik Hananel, Elizabeth David, Wladyslaw Gedroyc, Young-sun Kim, Matthias Matzko, Harsh Rastogi, Anne Roberts, Elizabeth Stewart, Robert Zurawin, Jaron Rabinovici

**Affiliations:** 1 Focused Ultrasound Foundation, Charlottesville, Virginia, United States; 2 Sunnybrook Health Sciences Centre, Toronto, Canada; 3 Imperial College London, London, United Kingdom; 4 Samsung Medical Center, Seoul, Republic of Korea; 5 Amper Kliniken AG, Dachau, Germany; 6 Indraprastha Apollo Hospital, Delhi, India; 7 University of California at San Diego, San Diego, California, United States; 8 Mayo Clinic, Rochester, Minnesota, United States; 9 Baylor College of Medicine, Houston, Texas, United States; 10 Sheba Medical Center, Tel Hashomer, Israel

## Background/introduction

In this work we will present our plan for an (MR)-guided, high-intensity focused ultrasound (FUS) uterine fibroid registry, with the goal of providing large-scale evidence on the safety and long-term efficacy of FUS for the treatment of symptomatic uterine fibroids.

## Methods

1,000 patients will be enrolled to the registry in multiple sites worldwide. Patient would get FUS treatment and be followed for a period of three years. Only FUS qualified patients and sites meeting registry treatment criteria would participate in the study.

## Results and conclusions

Treatment outcome and follow-up data will be collected by a contract research organization, (CRO), and analyzed for safety and efficacy. We will then compare our results to similar results in the literature. Subgroups will be selected and analyzed to address heterogeneity and usage of non-uniform treatment methods.

